# Decreased expression of STING predicts poor prognosis in patients with gastric cancer

**DOI:** 10.1038/srep39858

**Published:** 2017-02-08

**Authors:** Shushu Song, Peike Peng, Zhaoqing Tang, Junjie Zhao, Weicheng Wu, Haojie Li, Miaomiao Shao, Lili Li, Caiting Yang, Fangfang Duan, Mingming Zhang, Jie Zhang, Hao Wu, Can Li, Xuefei Wang, Hongshan Wang, Yuanyuan Ruan, Jianxin Gu

**Affiliations:** 1 Key Laboratory of Glycoconjugate Research Ministry of Public Health, School of Basic Medical Sciences, Fudan University, Shanghai, P.R. China; 2 Department of Biochemistry and Molecular Biology, School of Basic Medical Sciences, Fudan University, Shanghai, P.R. China; 3 Department of General Surgery, Zhongshan Hospital, Fudan University, Shanghai, P.R. China; 4 Institutes of Biomedical Sciences, Fudan University, Shanghai, P.R. China

## Abstract

STING (stimulator of interferon genes) has recently been found to play an important role in host defenses against virus and intracellular bacteria via the regulation of type-I IFN signaling and innate immunity. Chronic infection with *Helicobacter pylori* is identified as the strongest risk factor for gastric cancer. Thus, we aim to explore the function of STING signaling in the development of gastric cancer. Immunohistochemistry was used to detect STING expression in 217 gastric cancer patients who underwent surgical resection. STING protein expression was remarkably decreased in tumor tissues compared to non-tumor tissues, and low STING staining intensity was positively correlated with tumor size, tumor invasion depth, lymph mode metastasis, TNM stage, and reduced patients’ survival. Multivariate analysis identified STING as an independent prognostic factor, which could improve the predictive accuracy for overall survival when incorporated into TNM staging system. *In vitro* studies revealed that knock-down of STING promoted colony formation, viability, migration and invasion of gastric cancer cells, and also led to a defect in cytosolic DNA sensing. Besides, chronic *H. pylori* infection up-regulated STING expression and activated STING signaling in mice. In conclusion, STING was proposed as a novel independent prognostic factor and potential immunotherapeutic target for gastric cancer.

Gastric cancer remains the third leading cause of cancer-related mortality worldwide, although its incidence has decreased over the past six decades. The identified strongest risk factor for stomach cancer is chronic infection with *Helicobacter pylori*, a kind of genotoxic DNA pathogens^1^,^2^. Although current treatments such as surgery and chemotherapy have progressed, most patients are diagnosed at an advanced stage and still carry a dismal prognosis^3^,^4^. Therefore, molecular approaches are urgently needed in understanding tumor progression, in discovering novel biomarkers, and in determining effective therapies for use in clinical settings.

STING (stimulator of interferon genes), an endoplasmic reticulum localized protein, has recently been identified as one of the critical adaptors for cytosolic DNA sensing pathway^5^. It plays an important role in host defenses against virus and intracellular bacteria via the regulation of type-I IFN signaling and innate immunity^6^. STING can bind to cyclic dinucelotides such as cyclic di-AMP, cyclic di-GMP or cyclic di-GMP-AMP produced by virus or bacteria through its globular carboxy-terminal domain, thus facilitating its interaction with the cytosolic kinase TBK1 and inducing the activation of transcription factors IRF3 or STAT6. Then IRF3 or STAT6 translocate into nucleus to induce interferons and other cytokines^7^. Recently, several reports revealed the suppressive role of STING in tumorigenesis, including prostate cancer, colorectal carcinoma and melanomas^8^,^9^,^10^. However the role of STING in human gastric cancer remains largely unknown.

Considering the infection of *H. pylori* and the genomic DNA released by dying tumor cells^11^, STING might be involved in the tumorigenesis and progression of gastric cancer. The aim of this study was to evaluate the expression and function of STING in gastric cancer and elucidate its correlation with tumor development and prognosis.

## Results

### The expression of STING is down-regulated in gastric cancer

**Figure 1:**
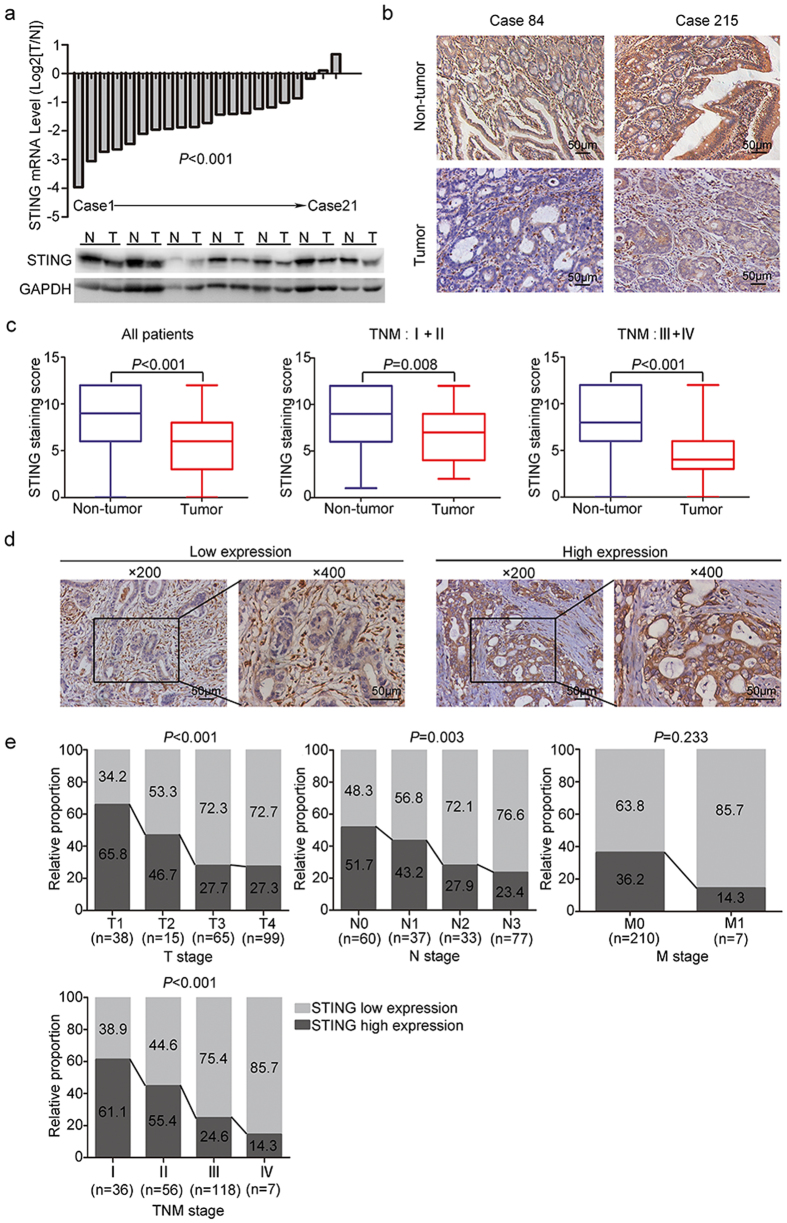
STING expression is down-regulated in gastric cancer and correlated with tumor progression. (**a**) The mRNA levels of STING in 21 cases of gastric cancer and paired adjacent non-tumor tissues were determined by real-time PCR analysis (top). And protein levels in 7 cases of gastric cancer and paired adjacent non-tumor tissues were determined by western blot analysis (bottom). N, adjacent non-tumor tissues; T, matched gastric cancer tissues. (**b**) Representative IHC staining of STING in tumor tissue and matched non-tumor tissue of gastric cancer patients. STING staining was mostly detected in cell cytoplasm. And STING was highly expressed in normal gastric epithelium, and was down-regulated in matched tumor tissues. The expression of STING was also detected in the surrounding stroma in some cases. (**c**) The staining score of tumor tissues comparing with normal tissues in all patients, TNM I-II subgroup, and TNM III-IV subgroup, respectively. (**d**) The representative low and high expression of STING in tumor tissues. (**e**) The relative proportion of patients with high STING expression is decreased with tumor progression in gastric cancer.

To explore the role of STING in the development of gastric cancer, the relative mRNA expression of STING was analyzed in 21 paired gastric cancer samples, and decreased expression of STING was observed in 90.5% cases (Fig. 1a). Western blot analysis revealed that STING protein levels were also remarkably reduced in tumor tissues compared with matched adjacent non-tumor tissues (Fig. 1a). Then a tissue microarray was employed to examine STING expression in 217 gastric cancer patients with different stages. The representative staining of STING in tumor tissues and pericarcinomatous tissues were shown in Fig. 1b. STING was mainly localized in the cell cytoplasm. Furthermore, we found that STING was mostly highly expressed in normal gastric epithelium, and was down-regulated in matched transformed tissues. The expression of STING was also detected in the surrounding stroma in some cases (Fig. 1b). Statistical analysis revealed that STING staining score in tumor cells was significantly lower than that in normal gastric epithelium (*P* < 0.001) in all patients (Fig. 1c). Moreover, STING staining levels were decreased in both TNM I–II and TNM III–IV subgroups, suggesting that reduced STING expression may manifest in early stage patients with gastric cancer (Fig. 1c).

### Correlations between intratumoral STING expression and clinicopathological features in gastric cancer patients

We next assessed the correlation between STING expression and clinicopathological features in gastric cancer patients. The high and low expression of intratumoral STING was determined by ROC curve analysis, and the representative images were shown in Fig. 1d. Chi-square analysis demonstrated that low expression of STING in gastric cancer was positively correlated with tumor size (*P* = 0.022), tumor invasion depth (*P* < 0.001), lymph mode metastasis (*P* = 0.003) and advanced TNM stage (*P* < 0.001) (Table 1). To better understand the role of STING in the progression of gastric cancer, the percentages of high and low expression of STING in different T stage, N stage, M stage and TNM stage were graphically displayed (Fig. 1e). Results demonstrated that the expression of STING was profoundly reduced with the progression of gastric cancer.

### Association of intratumoral STING expression with overall survival in gastric cancer patients

**Figure 2:**
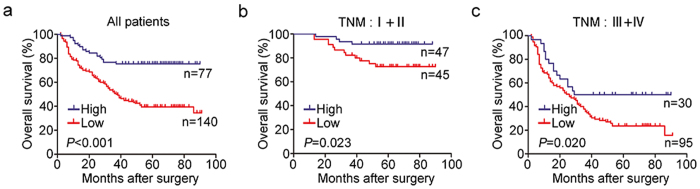
Kaplan-Meier analysis for overall survival of gastric cancer patients according to STING expression. The association of STING relative low and high expression in tumor tissues with overall survival was examined by Kaplan–Meier analysis, (**a**) in all patients; (**b**) in patients at TNM I+II stage; (**c**) in patients at TNM III+IV stage.

The association of intratumoral STING expression with overall survival was illustrated by Kaplan-Meier analysis. The results revealed that patients with STING low expression showed poorer overall survival than those with STING high expression (*P* < 0.001) (Fig. 2a). The overall survival rate of STING low expression group (39.3%) was nearly 2 times lower than the high expression group (75.3%). To further determine whether STING expression could stratify patients with different TNM stage, we grouped the TNM I + II and TNM III + IV as early and advanced stage disease, respectively. Similarly, low expression of STING was associated with shorter overall survival in both subgroups of gastric cancer patients (Fig. 2b,c).

### Prognostic factors for gastric cancer patients

**Figure 3:**
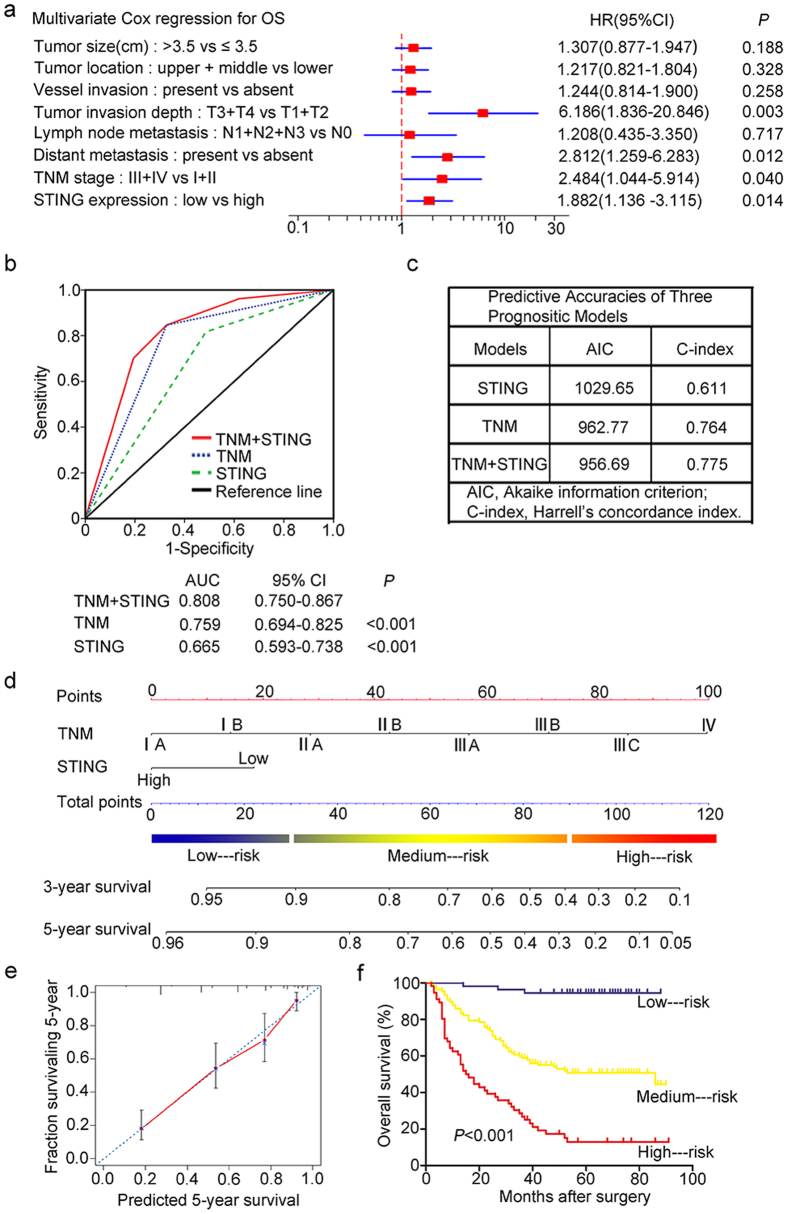
STING expression is an independent factor that could be combined with TNM stage for better prognostic prediction in gastric cancer patients. (**a**) Multivariate Cox analysis was performed to identify independent prognostic factors in patients with gastric cancer. (**b**) ROC analysis of the sensitivity and specificity for the predictive value of TNM model, STING expression model and the combined model. (**c**) The predictive accuracies of TNM staging, STING expression and the combined model were compared by AIC and C-index. Another prognostic predicting model nomogram was builted for overall survival in gastric cancer patients. (**d**) Nomogram was utilized to quantify the integrated effect of the proven independent prognostic factors for overall survival. (**e**) Calibration plot of the nomogram for 5-year survival. (**f**) Of all 217 patients, three groups were divided according to the total points in the nomogram which range of 0–30, 31–90, 91–120, was refined as low—, medium— and high—risk subgroup, respectively. Kaplan–Meier analysis was used to test the correlation of the risk with overall survival.

The univariate and multivariate analyses were performed to determine the prognostic factors for overall survival in gastric cancer patients. As shown in Table 2, patients’ tumor size (HR, 1.871; 95% CI, 1.266–2.767; *P* = 0.002), tumor location (HR, 1.828; 95% CI, 1.201–2.782; *P* = 0.005), vessel invasion (HR, 2.411; 95% CI, 1.507–3.855; *P* < 0.001), tumor invasion depth (HR, 3.947; 95% CI, 2.596–6.000; *P* < 0.001), lymph node metastasis (HR, 3.124; 95% CI, 2.076–4.701; *P* < 0.001), distant metastasis (HR, 37.03; 95% CI, 7.487–183.1; *P* < 0.001), TNM stage (HR, 4.880; 95% CI, 3.298–7.221; *P* < 0.001), and STING expression (HR, 2.599; 95% CI, 1.752–3.857; *P* < 0.001) were identified as risk factors that might affect the overall survival of gastric cancer patients. Further estimate by utilizing of multivariate Cox regression analysis demonstrated that tumor invasion depth (HR, 6.186; 95% CI, 1.836–20.846; *P* = 0.003), distant metastasis (HR, 2.812; 95% CI, 1.259–6.283; *P* = 0.012), TNM stage (HR, 2.484; 95% CI, 1.044–5.914; *P* = 0.040), and STING expression (HR, 1.882; 95% CI, 1.136–3.115; *P* = 0.014) were independent predictors for overall survival of gastric cancer patients (Fig. 3a).

### Improvement of the TNM staging prognostic model with STING expression

To establish a more accurate predictive model for outcomes of patients with gastric cancer, STING expression was combined with TNM staging system. The prognostic sensitive and specificity between STING expression with TNM staging system and each of them alone were compared by ROC analysis. Results illustrated that combination of STING expression and TNM staging system showed a significant better prognostic value (AUC, 0.808; 95% CI, 0.750–0.867) than TNM staging alone (AUC, 0.759; 95% CI, 0.694–0.825; *P* < 0.001), or STING expression alone (AUC, 0.665; 95% CI, 0.593–0.738; *P* < 0.001) (Fig. 3b). The AIC was reduced from 962.77 to 956.69, and the C-index was increased from 0.764 to 0.775 when the predictive model were created by combing STING expression with TNM staging system than the latter alone (Fig. 3c). All results above indicate that combination STING expression and TNM staging system could generate a more powerful predictive model for overall survival of patients with gastric cancer.

### Predictive nomogram for overall survival

In addition, a nomogram was constructed to integrate and quantify the proven independent prognostic factors for better outcome prediction. According to the multivariate analysis, TNM stage and STING expression were taken together in this nomogram to improve the accuracy (Fig. 3d). A higher total point indicated a poorer overall survival, and the predicted 3-year and 5-year survival was shown in our data. The performance characteristics for predicting 5-year survival was tested by the calibration plot (Fig. 3e). To further verify the prognostic significance of the nomogram, all patients were divided into three groups (low–risk, medium–risk and high–risk groups) according to the total points of each patient (Fig. 3d), and the result showed that the model could excellently stratified the risk of overall survival of gastric cancer patients (Fig. 3f).

### Knock-down of STING promotes the colony formation, viability, migration and invasion of gastric cancer cells

**Figure 4:**
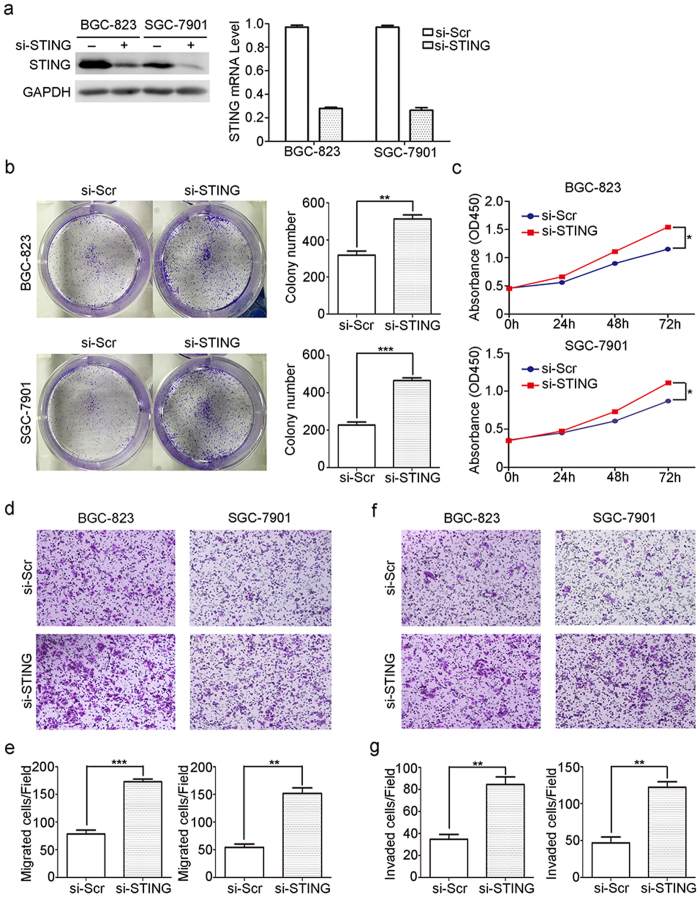
Knock-down of STING promotes the colony formation, viability, migration and invasion of gastric cancer cells. (**a**) The knock-down efficiency of STING siRNA in BGC-823 and SGC-7901 cells was monitored by western blot and real-time PCR. (**b**) Colony formation assays of BGC-823 and SGC-7901 cells transfected with STING siRNA or scrambled control siRNA. (**c**) The effects of STING knock-down on cell viabilities were examined by CCK8. (**d–g**) Transwell assays was employed to determine the influence of STING silencing on the migratory (**d,e**) and invasive (**f,g**) abilities in BGC-823 and SGC-7901 cells.

We next investigated the potential role of STING silencing in tumor characteristics in two kinds of gastric cancer cell lines BGC-823 and SGC-7901 (Fig. 4a). Colony formation and CCK8 assays revealed that knock-down of STING promoted the growth of BGC-823 and SGC-7901 cells *in vitro* (Fig. 4b,c). Moreover, transwell assays demonstrated that gastric cancer cells exhibited a higher migratory and invasive potential when STING expression was reduced (Fig. 4d–g). These data suggest the promotive role of reduced STING expression in cellular processes for tumorigenesis.

### Knock-down of STING inhibits the cytosolic DNA sensing and cGAMP-activating effects in gastric cancer cells

**Figure 5:**
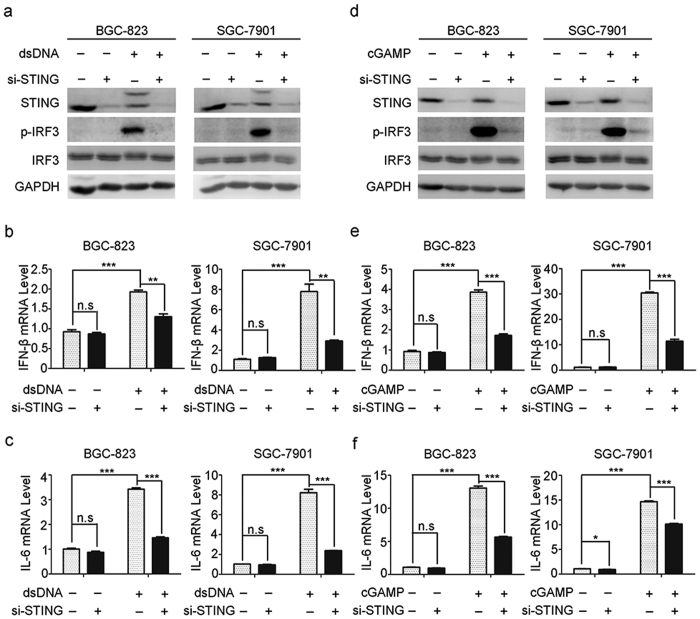
Knock-down of STING inhibits the cytosolic DNA sensing and cGAMP-activating effects in gastric cancer cells. (**a–c**) BGC-823 and SGC-7901 cells with or without STING depletion were transfected with dsDNA (4 μg/ml), and the expression level of STING and phosphorylation of IRF3 were then measured by western blot (**a**). IFN-β (**b**) and IL-6 (**c**) mRNA levels were measured by real-time PCR. (**d–f**) BGC-823 and SGC-7901 cells with or without STING depletion were stimulated with cGAMP (2 μg/ml), and the expression level of STING and phosphorylation of IRF3 were then measured by western blot (**d**). IFN-β (**e**) and IL-6 (**f**) mRNA levels were measured by real-time PCR.

STING has recently been identified as one of the critical adaptors for cytosolic DNA sensing, followed by the phosphorylation of IRF3 and subsequent production of type-I IFN and IL-6^12^. To explore whether STING expression levels correlated with its function, double-stranded DNA (dsDNA) was transfected into gastric cancer cells. Results demonstrated that dsDNA triggered the phosphorylation of IRF3 and the production of IFN-β and IL-6 (Fig. 5a–c). However, these effects were significantly blocked by siRNA-mediated knockdown of STING (Fig. 5a–c). Similar results were also observed upon the stimulation by cGAMP, a well-recognized agonist of STING signaling (Fig. 5d–f). In addition, we also found that dsDNA and cGAMP treatment decreased the protein levels of STING in gastric cancer cells (Fig. 5a,d), probably due to a negative-feedback control of STING activity which was also observed previously^13^.

### *Helicobacter pylori* infection activates STING signaling *in vivo*

**Figure 6:**
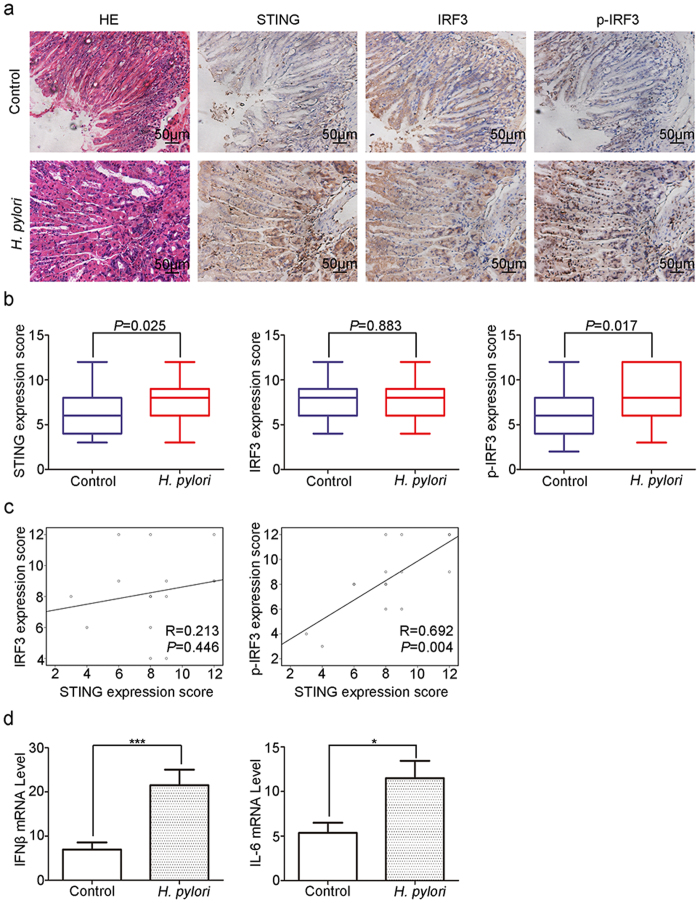
*Helicobacter pylori* infection activates STING signaling *in vivo*. Mouse model of chronic *H. pylori* infection was established as described in the “Materials and Methods”. (**a**) The expression pattern of STING and its downstream IRF3 phosphorylation in gastric epithelium were examined by IHC staining, and the representative images were shown. (**b**) The IHC staining scores of STING, IRF3 and p-IRF3 were compared between model group and control group. (**c**) Correlation analysis between STING expression and IRF3/p-IRF3 levels in *H. pylori*-infected mice. (**d**) The mRNA levels of IFN-β and IL-6 in stomach tissues were compared between model group and control group.

Chronic infection with *H. pylori*, a kind of genotoxic DNA pathogens, is well recognized as a risk factor for gastric cancer. Therefore, we next investigated the effect of *H. pylori* infection on STING signaling *in vivo*. After exposure to *H. pylori* for 20 weeks, STING expression, as well as IRF3 phosphorylation, was up-regulated in gastric epithelial cells, while little change was observed in the expression of IRF3 (Fig. 6a,b). Statistical analysis also confirmed the positive correlation of STING expression with IRF3 phosphorylation in the gastric epithelium of *H. pylori* infected mice (Fig. 6c). In addition, *H. pylori* infection also induced the mRNA expression of IFN-β and IL-6 in the stomach tissues of mice (Fig. 6d). These results indicate that *H. pylori* infection could activate STING signaling in stomach epithelium *in vivo*.

## Discussion

Traditional predictive model for outcomes of patients with gastric cancer mainly relies on TNM staging system including the information derived from tumor cell invasion depth, lymph node metastasis and distant metastasis. However, its ability to distinguish a subset of patients is limited due to the heterogeneity of tumor. Therefore, identifying new molecules associated with tumorigenesis in tumor cells would be helpful in understanding the progression of gastric cancer. STING has been extensively studied since the discovery of its participation in the detection of cytosolic DNA species^14^. Many kinds of DNA viruses including Hepatitis B Virus (HBV), Cytomegalovirus and Herpes Simplex Virus (HSV) as well as retroviruses such as Human Immunodeficiency Virus (HIV) can be negatively regulated by this innate immune pathway via the activation of interferon response^15^,^16^,^17^,^18^. Additionally, a diversity of bacterial infections and autoimmune diseases may also be associated with the modulation of the DNA sensing pathway^19^,^20^. Our present study is the first report to clarify the role of STING in the development of gastric cancer. And results indicated that loss of STING was positively associated with tumor progress which possibly due to its reduced function in DNA sensing, and STING could be regarded as a potential prognostic marker for gastric cancer patients.

Recently, accumulating studies have demonstrated the involvement of STING in immune surveillance in human cancers. Defective expression of STING was found in tumor tissues of colorectal carcinoma patients and peripheral blood mononuclear cells of HBV infected patients^9^,^21^. STING deficient mice also showed shorter survival when bearing with tumors^22^. Since gastric mucosa is the first line to defend foreign pathogens, high expression of STING in normal gastric epithelial cells (Fig. 1a,b) is likely to be required for in maintaining gastric homeostasis. Interestingly, *H. pylori* infection further up-regulated STING expression and activated STING signaling (Fig. 6), which implied that STING might play a role in *H. pylori*-induced chronic gastric inflammation. Nevertheless, we found that dsDNA and cGAMP stimulation could also activate STING signaling but caused the reduction in STING expression (Fig. 5a,d). In sight of these findings, we assume that direct DNA sensing, while activate STING signaling, might reduce STING expression probably in a negative feedback manner. And during the process of chronic *H. pylori* infection, other factors rather than DNA sensing could induce STING expression possibly for a sustained production of inflammatory cytokines. Though *H. pylori* infection has been recognized as a risk factor of gastric carcinogenesis, our data clearly indicated that STING expression was down-regulated in gastric cancer tissues (Fig. 1). Therefore, the decreased expression of STING in gastric cancer is unlikely to be directly caused by *H. pylori* infection, but might be critical for the tumor development through restraining immune surveillance.

The regulations of STING expression have also been studied. The ULK1 kinase which phosphorylated STING at S366, as well as E3 ubiquitin ligase TRIM30α and RNF5, were verified as negative regulators of STING by promoting its degradation^12^,^13^,^23^. Conversely, the processing body-associated protein LSm14A was required for the transcription and stability of STING^24^. In addition, analysis of STING SNPs data revealed that three non-synonymous SNPs, R71H-G230A-R293Q (HAQ), occur in approximately 20% of the population, but these variants did not obviously alter STING expression and/or stability^25^,^26^. Epigenetic processes are also likely to be the cause for reduced STING expression in gastric cancer patients. A recent study demonstrated the presence of considerable CpG islands within the STING promoter region^27^, and DNA hypermethylation might contribute to the suppressed STING expression in gastric cancer. These studies may provide clues to better understanding how the expression STING is deceased in tumor tissues.

Dynamic regulation of cytokines is the main way of STING to perform its function. Multifarious cytokines will be produced following the activation of STING signaling pathway, and subsequently recruit immune cells to microenvironment. Type-I IFNs have been identified as the major effector in STING-mediated anti-tumor immunity^28^. It is reported that type-I IFNs is essential for the maturation of CD11c^+^ CD8α^+^ DCs, and these DCs are critical for induction of tumor-reactive T-cell responses. Neutrophils were also shown to be polarized with type I interferons into anti-tumor N1 phenotype^29^,^30^. Type-I IFNs also contributes to the anti-tumor function of NK cells and macrophages^31^. Besides, other cytokines could also be regulated by STING, for example, IL-6 is responsible for the production of neutrophils, supports the growth of B cells and is antagonistic to regulatory T cells^32^. IL-12 leads to proliferation and activation of CD8^+^ T cells^33^, and CXCL10 may chemo-attract several kinds of immune cells^34^. Reduced STING expression rendered gastric cancer cells a defective function to produce type I interferon and other immune cytokines such as IL-6 after exposure to cytosolic DNA or its agonist cGAMP (Fig. 5). Hence, loss of STING may not only contribute to the *H. pylori* invasion and expansion in gastric mucosa, but also result in the defect of anti-tumor immunity in the process of gastric tumorigenesis. STING might also play a role in influencing the anti-tumor effects of checkpoint inhibitors such as CTLA4 and PD1^35^. Lei Jin *et al*. revealed a novel function of STING in regulating monocyte migration that was distinct from its role in activating cytokines production^36^. On the other hand, activation of STING could directly induce cell apoptosis and autophagy in malignant cells^37^,^38^, which might shape the inhibition of tumor growth.

In addition to its intratumoral staining, STING was detected in stroma in some cases of gastric cancer patients (Fig. 1b). By the present study, it remains to be determined whether STING expression in this place was associated with the progression of gastric cancer. However, extrinsic STING activity in tumor microenvironment apart from the tumor cells was also likely to play a critical role in the immune surveillance of tumor cells^39^. Overall, the profound molecular roles of STING signaling in gastric cancer remain far from being fully elucidated and need further investigation.

In conclusion, low STING expression was an independent and adverse predictor of overall survival in gastric cancer patients. And a more accurate predictive model for outcomes could be established by combining STING expression and TNM staging system. Knock-down of STING promoted colony formation, viability, migration and invasion in gastric cancer cells, and also led to a defect in cytosolic DNA sensing. Thus, STING might be a new biomarker for gastric cancer prognosis, and targeting STING with its agonist may provide novel approach for the immunotherapeutic treatment of gastric cancer.

## Materials and Methods

### Patient Samples

All of the methods were approved by the research medical ethics committee of Fudan University and were performed in accordance with the approved guidelines. Tumor and matched peritumoral specimens were obtained from 217 gastric cancer patients who underwent surgical resection without preoperative treatment from 2004 to 2008, at the Department of General Surgery, Zhongshan Hospital (Fudan University, Shanghai, China). Another independent group of 21 paired frozen gastric cancer and matched normal mucosa tissues was also collected at the Department of General Surgery, Zhongshan Hospital (Fudan University, Shanghai, China) in the year 2014. The diagnosis of gastric cancer was confirmed by pathologic examination. Patients’ clinicopathological characteristics, date of surgery, tumor stage, Lauren’s type, tumor location, surgical treatment methods, survival time, and other relevant clinicopathological data were obtained from hospital records. The use of human tissue samples and clinical data was approved by the Research Ethics Committee of Zhongshan Hospital. Informed consent was obtained from all patients.

### Animal Studies

Five to six-week-old male C57BL/6 mice were purchased from Shanghai Laboratory Animal Center of Chinese Academy Sciences and housed in a separate pathogen-free room. Animal care and experiments were performed in strict accordance with the Guide for the Care and Use of Laboratory Animals prepared by the National Academy of Sciences and published by the National Institutes of Health, and approved by the ethics committee of Fudan University. Mouse model of chronic *H. pylori* infection was established as described previously^40^. Briefly, each mouse was intragastrically administered with 1 × 10^8^ CFU *H. pylori* SS1 cells in 200 μl broth medium, while mice in the control group were intragastrically given with 200 μl broth medium, once every other day for 3 times. After 20 weeks, mice from both model group and control group were sacrificed and stomach tissues were collected for further analysis.

### Cell Lines

Human gastric cancer cell lines BGC-823 and SGC-7901 were obtained from Shanghai Cell Bank of Chinese Academy of Sciences (Shanghai, China). Both cells were cultured in Dulbecco’s modified Eagle’s medium replenished with 10% FBS (Gibco, Grand Island, NY, USA), and cultured at 37 °C in a humidified 5% CO2 incubator.

### Western Blot

Briefly, polyacrylamide gel electrophoresis was used to separate proteins which extracted from tissues or cells, and transferred onto polyvinylidene fluoride membranes. Membranes were incubated with primary antibodies including: STING (1:1000; Proteintech, Chicago, IL, USA), IRF3 (1:1000; Proteintech), p-IRF3 (1:1000; Cell Signaling, Boston, MA, USA), GAPDH (1:3000; Proteintech), and then with HRP-conjugated secondary antibody. At last, enhanced chemiluminescence assay was used to detect the reactions.

### Real-time PCR

Total RNA was purified from stomach tissues or cancer cells using TRIzol (Invitrogen, Carlsbad, CA, USA) according to the manufacturer’s instructions. The RNA was then processed for reverse transcription and quantitative PCR using a Takara RNA PCR Kit and SYBR Premix Ex Taq (Takara, Tokyo, Japan) in accordance with the manufacturer’s instructions. GAPDH was used as an internal control. The primers used were as follows: Human STING, sense: CCTGAGTCTCAGAACAACTGCC, anti-sense: GGTCTTCAAGCTGCCCACA-GTA; Human IFN-β, sense: AGGACAGGATGAACTTTGAC, anti-sense: TGATAGACATTAGCCAGGAG; Human IL-6, sense: AGACAGCCACTCACCT- CTTCAG, anti-sense: TTCTGCCAGTGCCTCTTTGCTG; Human GAPDH, sense: GAGTCAACGGATTTGGTCGT, anti-sense: TTGATTTTGGAGGGATCTCG; Mouse IFN-β, sense: TCCGAGCAGAGATCTTCAGGAA, antisense: TGCAACCA-CCACTCATTCTGAG; Mouse IL-6, sense: TCCAGTTGCCTTCTTGGGAC, anti-sense: GTGTAATTAAGCCTCCGACTTG; Mouse GAPDH, sense: GAGCGAGACCCCACTAACAT, anti-sense: TCTCCATGGTGGTGAAGACA.

### Tissue microarray construction and immunohistochemistry

Tissue microarray construction was carried out as previous described^41^. Primary antibody was used for IHC staining. In brief, the tissue microarrays were baked at 60 °C for 6 h, dewaxed in xylene, rehydrated through a gradient concentration and blocked the endogenous peroxidase activity by 3% hydrogen peroxide. After antigen retrieving by citrate buffer using microwave oven, the sections were incubated with the primary antibody including STING (1:200 dilution), IRF3 (1:100 dilution), p-IRF3 (1:100 dilution) at 4 °C overnight. Then, tissue sections were treated with Primary Antibody Amplifier Quanto and HRP Polymer Quanto (Thermo Scientific, Fremont, CA, USA). Finally, the sections were visualized by DAB solution and counterstained with haematoxylin. IHC staining score was assessed by two independent pathologists who were blinded to the patients’ clinicopathological data. The score for the extent of the IHC-stained area was set as 0 for <5%; 1 for 5–25%; 2 for 26–50%; 3 for 51–75%; and 4 for 76%–100% of tumor cells stained. The score for IHC intensity was also scaled as 0 for no IHC signal, 1 for weak, 2 for moderate, and 3 for strong. The final score used in the analysis was calculated by multiplying the extent score and intensity score, with a series of results ranging from 0 to 12. Values less than or equal to 6 were considered as the low expression, based on Receiver Operating Characteristic (ROC) analysis.

### siRNA, dsDNA transfection, and cGAMP treatment

The targeting sequence for STING siRNA was: 5′-CCTCATCAGTGGAATG-GAA-3′, according to a previous report^7^. Transfection of siRNA into gastric cancer cells was carried out using Lipofectamine 2000 (Invitrogen, Carlsbad, CA, USA) following the manufacturer’s instructions.

The dsDNA was prepared from equimolar amounts of the sense and antisense DNA oligonucleotide (sense strand sequence: 5′-TACAGATCTACTAGTGATCTAT-GACTGATCTGTACATGATCTACA-3′). The oligonucleotides in annealing buffer (Beyotime Biotechnology, Shanghai, China) were heated at 95 °C for 5 min and cooled to room temperature. Transfection of dsDNA into gastric cancer cells was also carried out using Lipofectamine 2000 (Invitrogen, Carlsbad, CA, USA) following the manufacturer’s instructions.

For cGAMP treatment, cGAMP (2 μg/ml) purchased from Invivogen (San Diego, CA, USA) was added to cells at 37 °C in a permeabilization buffer (50 mM HEPES, 100 mM KCl, 3 mM MgCl_2_, 85 mM sucrose, 0.1 mM DTT, 0.2% BSA, 1 mM ATP, 10 mg/ml digitonin) as described earlier^42^ and medium was replaced 30 min later. Then cells were harvested 3 hours post-treatment.

### Cell colony formation, viability and transwell assays

Gastric cancer cells were transfected as indicated. 48 h later, cells were used to perform the following assays. For colony formation assays, 1 × 10^3^ cells were seeded in 6-well plates. After culture for 10 days, cell colonies were fixed with paraformaldehyde (4%), stained with 0.1% crystal violet, and photographed using a digital camera. Colonies which contained 50 or more cells were counted. All experiments were performed at least three times.

For CCK8 assays, Cell Counting Kit-8 (DOJINDO, Kumamoto, Japan) was used to determine cell viability. Briefly, transfected cells were seeded in 96-well plates (3,000 cells per well), and cultured for the indicated times, then incubated with Cell Counting Kit-8 for 1 h. Absorbance was measured at 450 nm with a Universal Microplate Reader (Bio-Tek Instruments, Winooski, VT, USA). Three independent repeats were performed in all assays.

Transwell migration and invasion assays were performed in 12-well transwell plates (8 μm pore size) according to the manufacturer’s instructions (Corning, New York, NY, USA). For invasion assays only, the bottom of transwell chamber was coated with BD Matrigel Basement Membrane Matrix (BD Biosciences, San Diego, CA, USA). 1 × 10^5^ cells in basic culture medium without serum were added into the upper chamber, and the lower chamber was filled with culture medium containing 20% FBS as a chemo-attractant. Migration and invasion of cells were determined 24 h and 48 h later, respectively. Cells on the upper side of the chamber were removed from the surface of the membrane by scrubbing, and cells on the lower surface of the membrane were fixed with 4% paraformaldehyde and stained with 0.1% crystal violet. The number of infiltrating cells was counted in five randomly selected microscopic fields of each filter.

### Statistical Analysis

Analysis was performed with SPSS 22.0 (IBM Corporation, Armonk, NY, USA), GraphPad Prism 5 (GraphPad software, La Jolla, CA, USA), Stata 12.0 (Stata CorpLP, College Station TX, USA), and R software version 3.0.2 (R Foundation for Statistical Computing, Vienna, Austria). The relationships between clinical variables and STING expression was analyzed by Pearson χ^2^ test. Kaplan-Meier method was used to determine the overall survival and log-rank test was used to compare overall survival curve between different subgroups. Independent associations between overall survival and assessed clinicopathological predictors were evaluated by multivariate Cox proportional hazards regression models. Differences between two groups were examined by Student’s two-tailed *t*-test. Furthermore, R software was utilized to establish a nomogram and the predictive accuracy of this nomogram was tested by calibration plots. Correlation between two groups was analyzed using nonparametric Spearman’s r test. All statistical significance was set at two-sided and the *P* value was less than 0.05.

## Additional Information

**How to cite this article**: Song, S. *et al*. Decreased expression of STING predicts poor prognosis in patients with gastric cancer. *Sci. Rep.* **7**, 39858; doi: 10.1038/srep39858 (2017).

**Publisher's note:** Springer Nature remains neutral with regard to jurisdictional claims in published maps and institutional affiliations.

## Figures and Tables

**Table 1:** Relationship between STING expression and clinicopathological characteristics in patients with gastric cancer.

Factors	No.	STING expression		P-value
		Low	High	
		No. (%)	No. (%)	
Gender				
Male	144	97 (67.4)	47 (32.6)	0.219
Female	73	43 (58.9)	30 (41.1)	
Age (years)				
<60	102	64 (62.7)	38 (37.3)	0.608
≥60	115	76 (66.1)	39 (33.9)	
Tumor size (cm)				
Mean	3.8	4.1	3.4	**0.022**
Median	3.5	4.0	3.0	
IQR	2.0–6.0	2.0–6.0	1.5–5.0	
Tumor location				
Upper third	36	26 (72.8)	10 (27.2)	0.462
Middle third	37	25 (67.6)	12 (32.4)	
Lower third	144	89 (61.8)	55 (38.2)	
Lauren’s classification				
Intestinal	136	92 (67.6)	44 (32.4)	0.669
Diffuse	54	35 (64.8)	19 (35.2)	
Mixture	17	13 (76.5)	4 (23.5)	
Differentiation				
Poorly differentiated	183	121 (66.1)	62 (33.9)	0.252
Well differentiated	34	19 (55.9)	15 (44.1)	
Vessel invasion				
Absent	159	97 (61.0)	62 (39.0)	0.074
Present	58	43 (74.1)	15 (25.9)	
Tumor invasion depth				
T1	38	13 (34.2)	25 (65.8)	**<0.001**
T2	15	8 (53.3)	7 (46.7)	
T3	65	47 (72.3)	18 (27.7)	
T4	99	72 (72.7)	27 (27.3)	
Lymph node metastasis				
N0	60	29 (48.3)	31 (51.7)	**0.003**
N1	37	21 (56.8)	16 (43.2)	
N2	43	31 (72.1)	12 (27.9)	
N3	77	59 (76.6)	18 (23.4)	
Distant metastasis				
Absent	210	134 (63.8)	76 (36.2)	0.233
Present	7	6 (85.7)	1 (14.3)	
TNM stage				
I	36	14 (38.9)	22 (61.1)	**<0.001**
II	56	31 (55.4)	25 (44.6)	
III	118	89 (75.4)	29 (24.6)	
IV	7	6 (85.7)	1 (14.3)	

TNM = tumor node metastasis; IQR = inter quartile range. *P*-value < 0.05 marked in bold font shows statistical significant.

**Table 2:** Univariate Cox regression analysis of clinicopathological characteristics influencing the overall survival of gastric cancer patients.

Factors	Univariate		
	HR	95% CI	P-value
Gender			
Male *vs.* female	1.451	0.970–2.170	0.070
Age (years)			
≥ 60 *vs.* <60	1.258	0.854–1.853	0.246
Tumor size (cm)			
>3.5 *vs.* ≤3.5	1.871	1.266–2.767	**0.002**
Tumor location			
Upper + middle *vs.* lower	1.828	1.201–2.782	**0.005**
Lauren’s classification			
Diffuse + mixture *vs.* intestinal	1.365	0.898–2.076	0.145
Differentiation			
Poorly *vs.* well	1.038	0.611–1.762	0.891
Vessel invasion			
Present *vs.* absent	2.411	1.507–3.855	**<0.001**
Tumor invasion depth			
T3 + T4 *vs.* T1 + T2	3.947	2.596–6.000	**<0.001**
Lymph node metastasis			
N1 + N2 + N3 *vs.* N0	3.124	2.076–4.701	**<0.001**
Distant metastasis			
Present *vs.* absent	37.03	7.487–183.1	**<0.001**
TNM stage			
III + IV *vs.* I + II	4.880	3.298–7.221	**<0.001**
STING expression			
Low *vs.* high	2.599	1.752–3.857	**<0.001**

95% CI = 95% confidence interval; HR = hazard ratio; TNM = tumor node metastasis. *P*-value < 0.05 marked in bold font shows statistically significant.

## References

[b1] Torre L. A. . Global cancer statistics, 2012. CA: a cancer journal for clinicians 65, 87–108, doi: 10.3322/caac.21262 (2015). 25651787

[b2] Plummer M., Franceschi S., Vignat J., Forman D. & de Martel C. Global burden of gastric cancer attributable to Helicobacter pylori. International journal of cancer 136, 487–490, doi: 10.1002/ijc.28999 (2015). 24889903

[b3] Brenkman H. J., Haverkamp L., Ruurda J. P. & van Hillegersberg R. Worldwide practice in gastric cancer surgery. World journal of gastroenterology 22, 4041–4048, doi: 10.3748/wjg.v22.i15.4041 (2016). 27099448 PMC4823255

[b4] de Mestier L., Lardiere-Deguelte S., Volet J., Kianmanesh R. & Bouche O. Recent insights in the therapeutic management of patients with gastric cancer. Digestive and liver disease: official journal of the Italian Society of Gastroenterology and the Italian Association for the Study of the Liver, doi: 10.1016/j.dld.2016.04.010 (2016). 27156069

[b5] Xiao T. S. & Fitzgerald K. A. The cGAS-STING pathway for DNA sensing. Molecular cell 51, 135–139, doi: 10.1016/j.molcel.2013.07.004 (2013). 23870141 PMC3782533

[b6] Liu X. & Wang C. The emerging roles of the STING adaptor protein in immunity and diseases. Immunology 147, 285–291, doi: 10.1111/imm.12561 (2016). 26643733 PMC4754612

[b7] Wu J. . Cyclic GMP-AMP is an endogenous second messenger in innate immune signaling by cytosolic DNA. Science 339, 826–830, doi: 10.1126/science.1229963 (2013). 23258412 PMC3855410

[b8] Ho S. S. . The DNA Structure-Specific Endonuclease MUS81 Mediates DNA Sensor STING-Dependent Host Rejection of Prostate Cancer Cells. Immunity, doi: 10.1016/j.immuni.2016.04.010 (2016). 27178469

[b9] Xia T., Konno H., Ahn J. & Barber G. N. Deregulation of STING Signaling in Colorectal Carcinoma Constrains DNA Damage Responses and Correlates With Tumorigenesis. Cell reports 14, 282–297, doi: 10.1016/j.celrep.2015.12.029 (2016). 26748708 PMC4845097

[b10] Nakamura T. . Liposomes loaded with a STING pathway ligand, cyclic di-GMP, enhance cancer immunotherapy against metastatic melanoma. Journal of controlled release: official journal of the Controlled Release Society 216, 149–157, doi: 10.1016/j.jconrel.2015.08.026 (2015). 26282097

[b11] Hartung M. L. . H. pylori-Induced DNA Strand Breaks Are Introduced by Nucleotide Excision Repair Endonucleases and Promote NF-kappaB Target Gene Expression. Cell reports 13, 70–79, doi: 10.1016/j.celrep.2015.08.074 (2015). 26411687

[b12] Wang Y. . TRIM30alpha Is a Negative-Feedback Regulator of the Intracellular DNA and DNA Virus-Triggered Response by Targeting STING. PLoS pathogens 11, e1005012, doi: 10.1371/journal.ppat.1005012 (2015). 26114947 PMC4482643

[b13] Konno H., Konno K. & Barber G. N. Cyclic dinucleotides trigger ULK1 (ATG1) phosphorylation of STING to prevent sustained innate immune signaling. Cell 155, 688–698, doi: 10.1016/j.cell.2013.09.049 (2013). 24119841 PMC3881181

[b14] Ishikawa H., Ma Z. & Barber G. N. STING regulates intracellular DNA-mediated, type I interferon-dependent innate immunity. Nature 461, 788–792, doi: 10.1038/nature08476 (2009). 19776740 PMC4664154

[b15] Dansako H. . The cyclic GMP-AMP synthetase-STING signaling pathway is required for both the innate immune response against HBV and the suppression of HBV assembly. The FEBS journal 283, 144–156, doi: 10.1111/febs.13563 (2016). 26471009

[b16] Paijo J. . cGAS Senses Human Cytomegalovirus and Induces Type I Interferon Responses in Human Monocyte-Derived Cells. PLoS pathogens 12, e1005546, doi: 10.1371/journal.ppat.1005546 (2016). 27058035 PMC4825940

[b17] Parker Z. M., Murphy A. A. & Leib D. A. Role of the DNA Sensor STING in Protection from Lethal Infection following Corneal and Intracerebral Challenge with Herpes Simplex Virus 1. Journal of virology 89, 11080–11091, doi: 10.1128/JVI.00954-15 (2015). 26311879 PMC4621135

[b18] Guo H. . NLRX1 Sequesters STING to Negatively Regulate the Interferon Response, Thereby Facilitating the Replication of HIV-1 and DNA Viruses. Cell host & microbe 19, 515–528, doi: 10.1016/j.chom.2016.03.001 (2016). 27078069 PMC4833116

[b19] Dey B. . A bacterial cyclic dinucleotide activates the cytosolic surveillance pathway and mediates innate resistance to tuberculosis. Nature medicine 21, 401–406, doi: 10.1038/nm.3813 (2015). PMC4390473 25730264

[b20] Gao D. . Activation of cyclic GMP-AMP synthase by self-DNA causes autoimmune diseases. Proceedings of the National Academy of Sciences of the United States of America 112, E5699–5705, doi: 10.1073/pnas.1516465112 (2015). 26371324 PMC4620884

[b21] Karimi-Googheri M., Daneshvar H., Khaleghinia M., Bidaki R. & Kazemi Arababadi M. Decreased Expressions of STING but not IRF3 Molecules in Chronic HBV Infected Patients. Archives of Iranian medicine 18, 351–354, doi: 015186/AIM.005 (2015). 26058929

[b22] Anghelina D., Lam E. & Falck-Pedersen E. Diminished antiviral innate response to Adenovirus vectors in cGAS/STING deficient mice minimally impacts adaptive immunity. Journal of virology, doi: 10.1128/JVI.00500-16 (2016). PMC4907218 27076643

[b23] Zhong B. . The ubiquitin ligase RNF5 regulates antiviral responses by mediating degradation of the adaptor protein MITA. Immunity 30, 397–407, doi: 10.1016/j.immuni.2009.01.008 (2009). 19285439

[b24] Liu T. T. . LSm14A Plays a Critical Role in Antiviral Immune Responses by Regulating MITA Level in a Cell-Specific Manner. Journal of immunology, doi: 10.4049/jimmunol.1600212 (2016). 27183626

[b25] Yi G. . Single nucleotide polymorphisms of human STING can affect innate immune response to cyclic dinucleotides. PloS one 8, e77846, doi: 10.1371/journal.pone.0077846 (2013). 24204993 PMC3804601

[b26] Jin L. . Identification and characterization of a loss-of-function human MPYS variant. Genes and immunity 12, 263–269, doi: 10.1038/gene.2010.75 (2011). 21248775 PMC3107388

[b27] Xia T., Konno H. & Barber G. N. Recurrent loss of STING Signaling in Melanoma Correlates with Susceptibility to Viral Oncolysis. Cancer research, doi: 10.1158/0008-5472.CAN-16-1404 (2016). 27680683

[b28] Barber G. N. STING: infection, inflammation and cancer. Nature reviews. Immunology 15, 760–770, doi: 10.1038/nri3921 (2015). PMC5004891 26603901

[b29] Jablonska J., Leschner S., Westphal K., Lienenklaus S. & Weiss S. Neutrophils responsive to endogenous IFN-beta regulate tumor angiogenesis and growth in a mouse tumor model. The Journal of clinical investigation 120, 1151–1164, doi: 10.1172/JCI37223 (2010). 20237412 PMC2846036

[b30] Andzinski L. . Type I IFNs induce anti-tumor polarization of tumor associated neutrophils in mice and human. International journal of cancer 138, 1982–1993, doi: 10.1002/ijc.29945 (2016). 26619320

[b31] Ohkuri T. . STING contributes to antiglioma immunity via triggering type I IFN signals in the tumor microenvironment. Cancer immunology research 2, 1199–1208, doi: 10.1158/2326-6066.CIR-14-0099 (2014). 25300859 PMC4258479

[b32] Sin S. H. . Kaposi’s Sarcoma-Associated Herpesvirus Latency Locus Compensates for Interleukin-6 in Initial B Cell Activation. Journal of virology 90, 2150–2154, doi: 10.1128/JVI.02456-15 (2016). PMC4734016 26656696

[b33] Gravekamp C. & Chandra D. Targeting STING pathways for the treatment of cancer. Oncoimmunology 4, e988463, doi: 10.4161/2162402X.2014.988463 (2015). 26587334 PMC4635943

[b34] Liang D. . Activated STING enhances Tregs infiltration in the HPV-related carcinogenesis of tongue squamous cells via the c-jun/CCL22 signal. Biochimica et biophysica acta 1852, 2494–2503, doi: 10.1016/j.bbadis.2015.08.011 (2015). 26303640

[b35] Demaria O. . STING activation of tumor endothelial cells initiates spontaneous and therapeutic antitumor immunity. Proceedings of the National Academy of Sciences of the United States of America 112, 15408–15413, doi: 10.1073/pnas.1512832112 (2015). 26607445 PMC4687570

[b36] Jin L. . STING/MPYS mediates host defense against Listeria monocytogenes infection by regulating Ly6C(hi) monocyte migration. Journal of immunology 190, 2835–2843, doi: 10.4049/jimmunol.1201788 (2013). PMC3593745 23378430

[b37] Tang C. A. . Agonist-Mediated Activation of STING Induces Apoptosis in Malignant B Cells. Cancer research, doi: 10.1158/0008-5472.CAN-15-1885 (2016). PMC4873432 26951929

[b38] Watson R. O. . The Cytosolic Sensor cGAS Detects Mycobacterium tuberculosis DNA to Induce Type I Interferons and Activate Autophagy. Cell host & microbe 17, 811–819, doi: 10.1016/j.chom.2015.05.004 (2015). 26048136 PMC4466081

[b39] Corrales L. . Direct Activation of STING in the Tumor Microenvironment Leads to Potent and Systemic Tumor Regression and Immunity. Cell reports 11, 1018–1030, doi: 10.1016/j.celrep.2015.04.031 (2015). 25959818 PMC4440852

[b40] Peng P. . Decreased expression of Calpain-9 predicts unfavorable prognosis in patients with gastric cancer. Scientific reports 6, 29604, doi: 10.1038/srep29604 (2016). 27404891 PMC4941732

[b41] Chen L. . Loss of RACK1 Promotes Metastasis of Gastric Cancer by Inducing a miR-302c/IL8 Signaling Loop. Cancer research 75, 3832–3841, doi: 10.1158/0008-5472.CAN-14-3690 (2015). 26199092

[b42] Zhang Y. . The DNA sensor, cyclic GMP-AMP synthase, is essential for induction of IFN-beta during Chlamydia trachomatis infection. Journal of immunology 193, 2394–2404, doi: 10.4049/jimmunol.1302718 (2014). PMC4212656 25070851

